# Editorial: Connecting the dots in physiotherapy: reframing the role of the profession in the anthropocene

**DOI:** 10.3389/fpubh.2025.1697601

**Published:** 2025-09-24

**Authors:** Berta Paz-Lourido, Filip Maric

**Affiliations:** ^1^Department of Nursing and Physiotherapy, Health Research Institute of the Balearic Islands (IdISBa), University of the Balearic Islands, Palma de Mallorca, Spain; ^2^Department of Health and Care Sciences. Faculty of Health Sciences, UiT The Artic University of Norway, Tromsø, Norway

**Keywords:** physiotherapy, environment, planetary health, sustainability, climate change, public health

This Research Topic is the first comprehensive issue regarding the role that physiotherapists can play in the face of current environmental challenges, but also to recall the roots of the profession, linked to the environment in many diverse ways. It adds new data to the current debate about how to introduce planetary health and sustainability in healthcare education and practice (1). It shows that today's physiotherapists can be at the forefront of addressing the health impacts of climate change, advocating for sustainability and implementing a fundamentally revised vision of physiotherapy. Interdependence is a key concept for understanding how health, environment and society are interconnected across all levels and systems; it involves acknowledging complex planetary relationships, the situatedness of experience, and the partiality of knowledge (2). In this context, local nature-based interventions can be viewed as part of a global effort to enhance planetary health, with equity, sustainability, and transdisciplinary collaboration serving as guiding principles for action (3). Many other interventions can be developed from the various areas in which physiotherapists are involved, including research, education or management, requiring substantial policy changes at global, regional, national and local levels of a broader scope than the education or health sector (4). Physiotherapists around the world must take up the challenge to become sensitive and action-driven agents to address current environmental and social needs (5).

Higher education institutions are called to be active stakeholders regarding the Global Action Plan on Climate Change and Health approved at the Seventy-eighth World Health Assembly (6). Regarding physiotherapy, they should provide support for researchers, educators, students and clinicians to engage in sustainability initiatives, discover mentors in their area of interest, and receive funding for environmental physiotherapy projects. Physiotherapy leaders should promote sustainable practices to show that it is possible to minimize environmental impact, instill hope for present and future generations and engage with community partners in local and global initiatives. Physiotherapy students should have access to educational opportunities and pedagogical approaches to better understand the complex and multifactorial interactions between health and the environment (7). For this to happen, physiotherapy curricula should be framed within a broader awareness of the interdependent social and ecological drivers of planetary health, facilitating novel ways of promoting population health while considering environmental integrity (8). Voices from outside academia, such as those from vulnerable groups, rural partners, community organizations, and indigenous or native populations, should be heard as an integral part of this co-creation process in research (9), education (10), management and practice (11).

The Research Topic comprises nine contributions from authors affiliated with higher education and research institutions located in eight countries. These articles cover themes applicable to education, research and practice, questioning current knowledge, posing novel perspectives, raising conceptual analysis and illustrating the possibilities for transferability to different contexts.

Regarding physiotherapy education, the work developed by Stone et al. describes the integration of environmental physiotherapy education into the physiotherapy curricula with the aim of developing holistic healthcare perspectives in students that will strengthen future physiotherapy practice. The article authored by Fradejas et al. clarifies how physiotherapy students involved in intergenerational service-learning programs benefit by promoting physical activity, wellbeing and social interaction within the community. The research by Swärdh et al. highlights the perspectives of physiotherapy educators, indicating that a disconnection remains between educational attitudes and actions. A key priority, then, is to offer new perspectives on professional identity and continuing professional development within the context of sustainable development. The proposal by Paz-Lourido and Ribeiro-Chaves emphasizes the need for the regeneration of the educational curriculum in physiotherapy to facilitate a better alignment between planetary health and service-learning as a pedagogical approach.

Still connected to education, but moving toward practice, the work done by Ólafsdóttir and Petursdottir highlights the potential for physiotherapists to make significant contributions to the healthcare sector's sustainability goals, not only by contributing to reducing the healthcare sector's carbon footprint but also by promoting sustainable health behaviors. In turn, Persson et al. argued that multidisciplinary teams are essential for achieving sustainability, optimizing patient functioning, and preventing disease, promoting patient empowerment, implementing lean pathways, using low-carbon alternatives, and ensuring efficient resource use. The article by Doran-Sherlock et al. highlights some of the key challenges in the nature-based health and therapy literature, stating that the quality of many studies in the broader nature-based health and therapy literature is poor so far and has often been conducted within a reductionist biomedical framework. A list of considerations is provided to remedy this status quo and advance nature-based health and therapy.

The review by Svensson et al. mapped existing research comparing physiotherapy to pharmaceuticals, with a specific focus on whether these studies address aspects of sustainable development. It shows that studies are lacking that evaluate outcomes from a sustainable development perspective which could contribute to knowledge about how physiotherapy can be a low-carbon, resource-efficient alternative to pharmaceuticals. Finally, the perspective raised by Sturm et al. points to the need to move beyond an isolated profession-centric approach when tackling the existing, concerning issues in child health worldwide. It demonstrates how physiotherapists can contribute to improved child health by incorporating risky play into physiotherapy theory and practice.

In the light of these works, it is urgent to connect the dots and reframe physiotherapy education, research and practice. This means embedding planetary health and considering research from various epistemological traditions, promoting innovative experiential pedagogies and strengthening community engagement in physiotherapy. This would facilitate entirely novel professional perspectives, and create spaces for future studies, theoretical frameworks and practical guidelines. It is necessary to address the barriers of lack of knowledge, material resources, and funding, and potentiate motivators such as education and guidance on the environment, physiotherapy and health.
